# Supplementary material for: WordCommentsAnalyzer: A windows software tool for qualitative research
Source: F1000Res. 2018 Sep 4;7:536. Originally published 2018 May 3. [Version 2] doi: 10.12688/f1000research.14819.2 (PMC6137411; doi:10.12688/f1000research.14819.2)
Supplement: Supplementary file 3 [file f1000research-7-17614-s0002.tgz › b2a79496-4ada-47c4-9682-a99ce867836d.zip!/Eight Iranian Health Journals/Journal of Research in Rehabilitation Sciences _8.docx]

© Journal of Research in Rehabilitation Sciences . This work is licensed under CC BY-NC 4.0 (https://creativecommons.org/licenses/by-nc/4.0/).

Volume & Issue: دوره 12، شماره 3: 1395

Title: تحلیل آکوستیکی زمان‌بندی گفتار در افراد مبتلا به دیزآرتری فلاسید

http://jrrs.mui.ac.ir/index.php/jrrs/article/view/2618

مقدمه: دیزآرتری (Dysarthria) فلاسید گروهی از اختلالات حرکتی گفتار است. مشخصه‌های گفتاری اصلی این اختلال شامل ضعف و کاهش تون عضلانی، کاهش سرعت، دامنه و دقت حرکات گفتاری می‌باشد. الگوهای آکوستیکی گفتار در این افراد نقایصی را در زمینه کنترل حرکتی نشان می‌دهد که می‌توان به مشکلات در زمان‌بندی، هماهنگی حرکات تولیدی و کنترل حنجره‌ای اشاره کرد. مطالعه حاضر با هدف بررسی زمان‌بندی گفتار از طریق تحلیل آکوستیکی انتقال فورمنت دوم در افراد مبتلا به دیزآرتری فلاسید انجام شد. مواد و روش‌ها: اين مطالعه توصیفی- تحلیلی از نوع مقطعی و مورد- شاهدی بود. افراد مورد مطالعه را 10 فرد مبتلا به دیزآرتری فلاسید خفیف تا متوسط و 10 فرد طبیعی تشکیل داد. پس از قرارگیری آزمودنی در محیط مناسب، سیگنال‌های آکوستیکی مربوط به کلمات هدف که شامل انسدادی‌های واک‌دار و بی‌واک بود، جمع‌آوری و ضبط شد. سپس با استفاده از نرم‌افزار Praat، اسپکتروگرام هر کلمه به منظور تعیین انتقال فورمنت دوم مورد بررسی قرار گرفت. یافته‌ها: افراد مبتلا به دیزآرتری فلاسید خفیف تا متوسط نسبت به افراد طبیعی، انتقال فورمنت طولانی‌تری داشتند (به استثنای /pa/). همچنین، مبتلایان به دیزآرتری نسبت به افراد گروه شاهد، در انتقال فورمنت دوم در انسدادی‌های دولبی و دندانی واک‌دار و بی‌واک، تفاوت معنی‌داری را نشان دادند (050/0 > P). نتیجه‎گیری: افزایش انتقال فورمنت دوم در مبتلایان به دیزآرتری فلاسید خفیف تا متوسط، نشان داد که در این افراد نقایص در یکپارچه سازی، زمان‌بندی حرکات و اجرای تولیدی برای سگمنت هدف وجود دارد. همچنین، کاربرد بالینی نتایج به دست آمده این است که همخوان‌های متفاوت، حساسیت‌های مختلفی به مشکلات کنترل حرکتی گفتار نشان می‌دهند. آسیب‌شناسان گفتار و زبان باید توجه ویژه‌ای به دیرش انتقال فورمنت دوم در همخوان‌های دولبی و دندانی و میزان تغییر آن در ارزیابی‌های آکوستیکی افراد مبتلا به دیزآرتری فلاسید داشته باشند.

Title: رابطه بین تعمیم‌پذیری درمان مشکلات نامیدن با سطوح آسیب‌دیده در نامیدن

http://jrrs.mui.ac.ir/index.php/jrrs/article/view/2651

مقدمه: علامت اصلی زبان‌پریشی، اختلال در نامیدن است. اگرچه در مطالعات مختلف مشخص شده است که مداخلات درمانی در بهبود مشکلات نامیدن مؤثر می‌باشد، اما بیشتر این مداخلات منجر به تعمیم در درمان نمی‌شود. عوامل دخیل در عدم تعمیم درمان مشخص نیست. ممکن است سطوح آسیب دیده در فرایند بازیابی واژه بر این امر تأثیر بگذارد. به منظور مشخص نمودن این ارتباط، مطالعه حاضر انجام گرفت. در این مطالعه از درمان‌هایی که سطوح آسیب دیده بازیابی واژه را هدف قرار می‌دهند، استفاده گردید. مواد و روش‌ها: روش انجام مطالعه حاضر به صورت طرح مورد- منفرد متقاطع بود. چهار فرد زبان‌پریش که شکایت اصلی آن‌ها، مشکل در نامیدن بود، انتخاب شدند. هر بیمار درمان‌های تحلیل مشخصات معنایی و تحلیل مؤلفه‌های واجی را دریافت نمود. برای هر بیمار یک‌سری کلمات غیر آموزشی در نظر گرفته شد که تعمیم با صحت درستی نامیدن در این کلمات مورد بررسی قرار گرفت. یافته‌ها: اگرچه هر دو رویکرد درمانی منجر به بهبود توانایی نامیدن کلمات غیر آموزشی شد، اما تعمیم در شرکت کنندگان دارای آسیب واجی به دنبال درمان واج‌‌شناختی و در شرکت کنندگان دارای آسیب معنایی به دنبال درمان معنایی به طور قابل ملاحظه‌ای بیشتر بود. نتیجه‎گیری: توانایی پیش‌بینی الگوی تعمیم به دنبال یک رویکرد درمانی خاص، دارای اهمیت بالینی است. پیشنهاد می‌شود که بر اساس یک مدل روان زبان‌شناختی، ابتدا سطح آسیب بازیابی واژه تعیین شود و متناسب با آسیب، درمانی که سطح شکست را هدف قرار می‌دهد، استفاده گردد.

Title: انرژي مکانيکي اندام تحتاني زنان مبتلا به شدت‌های مختلف استئوآرتريت زانو در حين راه رفتن: مطالعه مورد- شاهدی

http://jrrs.mui.ac.ir/index.php/jrrs/article/view/2656

مقدمه: استئوآرتريت زانو (Knee osteoarthritis یا KOA) يکي از شايع‌ترين اختلالات عضلاني- اسکلتي در جامعه می‌باشد. سنجش مصرف انرژي حين راه رفتن در افراد دچار اختلالات عضلاني- اسکلتي، مي‌تواند مستندات عيني از ميزان ناتواني اين افراد و تأثیر درمان‌هاي مختلف را در اختيار پژوهشگران قرار دهد. هدف از انجام مطالعه حاضر، بررسي تأثير شدت‌هاي مختلف استئوآرتريت زانو بر ميزان انرژي مکانيکي اندام تحتانی بود. مواد و روش‌ها: 48 زن 40 تا 70 ساله بر اساس شواهد راديولوژيک زانوهاي خود، در سه گروه استئوآرتريت خفيف، متوسط و شديد و يک گروه سالم تقسيم‌بندی شدند. در آزمايشگاه، شرکت کنندگان با سرعت دلخواه براي 5 ثبت موفق با استفاده از سيستم آناليز حرکت راه رفتند و مدل‌هاي سه بعدي سگمان‌ها در نرم‌افزار Visual3D ساخته شد. انرژي مکانيکي اندام تحتاني با استفاده از اين مدل‌ها به دست آمد. یافته‌ها: تفاوت معنی‌داري بین گروه‌هاي مورد بررسي از لحاظ سن (001/0 ≥ P) و سرعت راه رفتن (001/0 ≥ P) وجود داشت. با استفاده از تحليل واريانس و آزمون‌هاي تعقيبي، تفاوت‌هايي بين انرژی مکانیکی گروه‌هاي مورد مطالعه به ويژه گروه استئوآرتريت متوسط و گروه سالم مشاهده شد. بنابراين، مدل‌هاي خطي تک متغيره جهت کنترل اثر سن و سرعت راه رفتن و مدل‌هاي خطي دو متغيره براي کنترل هم‌زمان اثر سن و سرعت ساخته شد. اين مدل‌ها نشان داد که شدت استئوآرتريت، تأثير معنی‌داري بر انرژي مکانيکي اندام تحتاني مبتلايان به استئوآرتريت زانو نداشت. نتیجه‎گیری: به نظر مي‌رسد که افزايش انرژي مکانيکي در مبتلايان به استئوآرتريت زانو، با افزايش شدت بيماري تشديد مي‌شود. اين مسأله نتيجه مستقيم افزايش شدت درگيري مفصل نيست، بلکه نتيجه غير مستقيم بالاتر بودن سن و کمتر بودن سرعت راه رفتن افراد مبتلا به درجات پيشرفته‌تر بيماري مي‌باشد.

Title: بررسی مقدماتی تأثیر راه رفتن بر روی شیب در تحمل عضلات و ناتوانی بیماران مبتلا به کمردرد مزمن: کارآزمایی بالینی

http://jrrs.mui.ac.ir/index.php/jrrs/article/view/2678

مقدمه: در بیماران مبتلا به کمردرد مزمن، راه رفتن به عنوان یک تمرین درمانی ساده، سالم، کم‌هزینه و در دسترس، به بررسی بیشتری نیاز دارد. هدف از انجام مطالعه حاضر، بررسی تأثیر راه رفتن بر روی تردمیل با شیب 10 درصد، بر تحمل عضلات، درد، ناتوانی و ترس از حرکت در بیماران مبتلا به کمردرد مزمن غیر اختصاصی بود. مواد و روش‌ها: شرکت کنندگان به دو گروه شاهد (درمان استاندارد فیزیوتراپی و تردمیل با شیب پایه، 6 نفر) و مداخله (درمان استاندارد فیزیوتراپی و تردمیل با شیب 10 درصد، 5 نفر) تقسیم شدند. بیماران سه بار در هفته به مدت چهار هفته تحت درمان قرار گرفتند. تحمل عضلات خلف تنه، ترس از فعالیت، درد و ناتوانی به ترتیب با آزمون Sorensen تغییر یافته و پرسش‌نامه‌های ترس از حرکت، درد و Oswestry قبل از مطالعه، بلافاصله پس از مطالعه و دو ماه پس از درمان اندازه‌گیری شد. یافته‌ها: تفاوت بین گروهی در متغیر تحمل عضلات خلف تنه پس از درمان (01/0 = P) و در زمان پیگیری (04/0 = P) وجود داشت. در گروه مداخله، تغییرات در متغیرهای تحمل عضلات خلف تنه (53/0 = P)، درد (26/0 = P)، ناتوانی (50/0 = P) و ترس از حرکت (90/0 = P) معنی‌دار نبود. در گروه شاهد نیز تغییرات معنی‌داری در متغیرهای تحمل عضلات خلف تنه (19/0 = P)، درد (57/0 = P)، ناتوانی (22/0 = P) و ترس از حرکت (23/0 = P) مشاهده نشد. بین دو گروه در متغیرهای درد (14/0 = P)، ناتوانی (71/0 = P) و ترس از حرکت (27/0 = P) تفاوت معنی‌داری وجود نداشت. نتیجه‎گیری: تفاوت معنی‌داری در افزایش تحمل عضلات تنه بین گروه‌های شیب 10 درصد و پایه وجود دارد. به نظر می‌رسد که راه رفتن بر روی تردمیل در گروه شیب پایه مؤثرتر است.

Title: بررسی اثر خستگی عملکردی بر ارتفاع پرش عمودی در زنان و مردان سالم

http://jrrs.mui.ac.ir/index.php/jrrs/article/view/2688

مقدمه: پرش عمودی یکی از فعالیت‌های پایه ورزشی و آزمون بسیار شایعی در حیطه توان‌بخشی و ورزش است که به کمک آن می‌توان عملکرد و توان اندام تحتانی و سیستم عضلانی- اسکلتی بدن را بررسی نمود. از آن‌جایی‌ که خستگی یکی از عوامل مؤثر در افزایش خطر ابتلا به آسیب است، هدف از انجام مطالعه حاضر، بررسی تفاوت اثر خستگی در زنان و مردان حین پرش عمودی بود. مواد و روش‌ها: در این مطالعه مقطعی، 18 زن و 14 مرد، پرش عمودی را قبل و بعد از پروتکل خستگی عملکردی اجرا کردند. پروتکل خستگی عملکردی این طرح شامل 5 مرحله پرش بود. پرش یک نوع سیکل کشش- کوتاه شدن عضلانی است که می‌تواند فعالیت طبیعی عضلات را حین فعالیت‌های روزمره و ورزشی شبیه‌سازی کند. متغیر مورد بررسی این تحقیق، ارتفاع پرش عمودی بود. داده‌های مطالعه با استفاده از آزمون تحلیل واریانس با اندازه‌گیری مکرر بررسی گردید. یافته‌ها: بین دو گروه اثر متقابلی مشاهده نشد. ارتفاع پرش در مردان در هر دو شرایط قبل و بعد از خستگی بیشتر از زنان بود. همچنین، خستگی در هر دو گروه منجر به کاهش مقدار ارتفاع پرش شد. نتیجه‎گیری: نتیجه اصلی مطالعه حاضر، پاسخ مشابه مردان و زنان به خستگی بود. به نظر می‌رسد که خستگی عضلانی مانع از فعالیت مناسب عضلات برای کاهش شوک ناشی از ضربه و در نتیجه، انتقال بیشتر نیروی عکس‌العمل زمین به سمت مفاصل بالاتر و آسیب استخوانی می‌شود.

Title: بررسی اثر برنامه تمرینی حس عمقی گردن بر شاخص‌های کینماتیکی سر و گردن در برابر اغتشاش خارجی میان ورزشکاران کاراته‌کای نخبه مرد

http://jrrs.mui.ac.ir/index.php/jrrs/article/view/2679

مقدمه: وجود اغتشاشات خارجی تکراری و یا با شدت بالا، از جمله مکانیسم‌های اصلی آسیب‌های ستون فقرات گردن و سر در ورزش‌های برخوردی مانند کاراته می‌باشد. سیستم عصبی با به کارگیری استراتژی‌های ثبات پوسچرال، سعی بر به حداقل رساندن اثر اغتشاشات در بدن دارد. هدف از انجام تحقیق حاضر، بررسی اثر تمرینات حس عمقی گردن بر استراتژی‌های ثبات پوسچرال سر و گردن جهت کاهش نوسانات این نواحی در نتیجه اغتشاشات خارجی در ورزشکاران کاراته‌کا بود. مواد و روش‌ها: 25 ورزشکار داوطلب مرد کاراته‌کا به طور تصادفی در دو گروه شاهد (12 نفر) و تجربی (13 نفر) قرار گرفتند. جهت اندازه‌گیری شاخص‌های کینماتیکی سر و گردن در برابر اغتشاش خارجی، از دستگاه آنالیز حرکت استفاده گردید. داده‌ها با استفاده از آزمون‌های Independent t، Paired t و تحلیل کوواریانس در نرم‌افزارهای CORTEX، MATLAB و SPSS مورد تجزیه و تحلیل قرار گرفت. 05/0 > P به عنوان سطح معنی‌داری در نظر گرفته شد. یافته‌ها: تفاوت معنی‌داری از نظر جابه‌جایی زاویه‌ای (گروه تجربی 96/0 ± 69/2 و گروه شاهد 87/0 ± 44/3؛ 009/0 = P) و شتاب زاویه‌ای (گروه تجربی 92/4 ± 07/14 و گروه شاهد 14/9 ± 54/33؛ 001/0 > P) سر در برابر اغتشاش، بین دو گروه در پس‌آزمون وجود داشت. نتیجه‎گیری: تمرینات حس عمقی ناحیه گردن می‌تواند موجب افزایش کارایی استراتژی‌های ثبات پوسچرال و کاهش میزان نوسانات ناحیه سر و گردن در مواجهه با اغتشاشات خارجی در ورزشکاران کاراته‌کا شود.

Title: قانون حمایت از حقوق افراد با ناتوانی: مروری بر چالش‌ها و ناهمانندی‌ها در اجرا و دستاوردهای آن در چند کشور

http://jrrs.mui.ac.ir/index.php/jrrs/article/view/2691

مقدمه: در گزارش سازمان بهداشت جهانی، تعداد افراد دارای انواع ناتوانی در جهان که بزرگ‌ترین اقلیت غیر نژادی به ‌شمار می‌روند، بیش از یک میلیارد نفر برآورد شده است که 20 درصد از آن‌ها را فقیرترین افراد جهان تشکیل می‌دهند. پژوهش حاضر با هدف مرور و مقایسه‌ کارکرد کشورهای مورد مطالعه در چگونگی‌ اجرا و پیشبرد پیمان قانون حقوق افراد با ناتوانی (سازمان ملل متحد) انجام شد. مواد و روش‌ها: کشورهای مورد پژوهش شامل آمریکا، انگلستان، فرانسه، روسیه، ایران و برخی کشورهای در حال توسعه بود. جمع‌آوری اطلاعات مورد نیاز درباره‌ این کشورها با جستجوی گسترده در پایگاه‌های علمی‌ معتبر داخلی و جهانی انجام گرفت. یافته‌ها: یافته‌های پژوهش در بخش‌هایی شامل توصیف برخی ویژگی‌های افراد با ناتوانی در کشورهای مورد مطالعه و بررسی‌ وضعیت حقوق افراد با انواع ناتوانی در زمینه‌های آموزش و پرورش، به ‌کارگماری، دسترسی‌پذیری به خدمات و مناسب‌سازی فضای زندگی‌ اجتماعی- شهری ارایه شده‌ است. کارکرد کشورهای مورد مطالعه در اجرای این قانون، نشان‌ دهنده‌ شکاف زیادی بین آن‌ها بود و هر یک از این کشورها با چالش‌های ویژه‌ای در پیشبرد آن روبه‌رو هستند. نتیجه‎گیری: دستیابی به اهداف قانون حقوق افراد با انواع ناتوانی، نه تنها نیازمند تصویب و اجرای قوانین و اصلاح ساختارها است، بلکه در گرو تغییر در نگرش‌های اجتماعی و فرهنگی برای پذیرش و احترام به کرامت انسانی این افراد نیز می‌باشد تا همراه با بسترسازی‌های محیطی، مناسب‌سازی‌ برنامه‌های آموزشی- حرفه‌ای و مدیریت بودجه و امکانات، مؤثر واقع شود.

Title: وسعت تحقیقاتی معنویت در متون کاردرمانی: بررسی مروری حیطه‌ای

http://jrrs.mui.ac.ir/index.php/jrrs/article/view/2594

مقدمه: معنویت، افراد را به سمت جستجوی معنا و بیان هدف در زندگی سوق می‌دهد. کاردرمانی، حرفه‌ای کل‌نگر می‌باشد و معتقد است که تمام جوانب تجربه شده توسط انسان از جمله جوانب جسمی، روانی، اجتماعی و معنوی، جزء ابعاد مهم سلامتی به شمار می‌روند. با این حال، توافق عمومی بر این است که تاکنون متون کاردرمانی به خوبی به مفهوم معنویت نپرداخته‌اند. بنابراین، هدف از انجام مطالعه حاضر، ارایه گزارشی از وسعت و طبیعت تحقیقاتی معنویت در کاردرمانی و یافتن شکاف‌های موجود در این حیطه بود. مواد و روش‌ها: این مطالعه به روش مروری حیطه‌ای انجام شد. بدین منظور، پایگاه‌های مختلف زیستی- پزشکی و توان‌بخشی با استفاده از کلید واژه‌های مرتبط، مورد جستجو قرار گرفت. بازه زمانی جستجو از مبنای زمانی پایگاه اطلاعاتی تا ماه اکتبر سال 2015 بود. یافته‌ها: در نهایت، 45 متن وارد این مرور گردید. این متون شامل مقالات اصیل (0/49 درصد)، مقالات مروری و آموزشی (6/24 درصد)، کتاب و فصل کتاب (8/8 درصد)، مدل و تئوری (8/8 درصد) و نقطه نظر (8/8 درصد) بود. نتیجه‎گیری: معنویت یکی از مسایل مورد توجه متون کاردرمانی می‌باشد، اما تعداد محدود متون، نشان دهنده آن است که محققان کمی به پژوهش در این حیطه پرداخته‌اند. در مجموع، نتایج پژوهش حاضر نشان داد که تعداد مطالعات صورت گرفته در حیطه معنویت در کاردرمانی محدود و اغلب در مورد نقش معنویت و نظر کاردرمانگران راجع به آن بوده است و کمتر به مفهوم معنویت، تعریف آن، مداخلات موجود در این حیطه و روند مواجه شدن با نیازهای معنوی مراجعان اشاره شده است.
